# Privacy-Preserving Glycemic Management in Type 1 Diabetes: Development and Validation of a Multiobjective Federated Reinforcement Learning Framework

**DOI:** 10.2196/72874

**Published:** 2025-07-04

**Authors:** Fatemeh Sarani Rad, Juan Li

**Affiliations:** 1 Computer Science Department, North Dakota State University, 1320 Albrecht Blvd, Fargo, ND, 58105, United States, 1 7012319662

**Keywords:** reinforcement learning, federated learning, federated reinforcement learning, multiobjective optimization, diabetes management, blood glucose control, privacy-preserving artificial intelligence, reward shaping, AI

## Abstract

**Background:**

Effective diabetes management requires precise glycemic control to prevent both hypoglycemia and hyperglycemia, yet existing machine learning (ML) and reinforcement learning (RL) approaches often fail to balance competing objectives. Traditional RL-based glucose regulation systems primarily focus on single-objective optimization, overlooking factors such as minimizing insulin overuse, reducing glycemic variability, and ensuring patient safety. Furthermore, these approaches typically rely on centralized data processing, which raises privacy concerns due to the sensitive nature of health care data. There is a critical need for a decentralized, privacy-preserving framework that can personalize blood glucose regulation while addressing the multiobjective nature of diabetes management.

**Objective:**

This study aimed to develop and validate PRIMO-FRL (Privacy-Preserving Reinforcement Learning for Individualized Multi-Objective Glycemic Management Using Federated Reinforcement Learning), a novel framework that optimizes clinical objectives—maximizing time in range (TIR), reducing hypoglycemia and hyperglycemia, and minimizing glycemic risk—while preserving patient privacy.

**Methods:**

We developed PRIMO-FRL, integrating multiobjective reward shaping to dynamically balance glucose stability, insulin efficiency, and risk reduction. The model was trained and tested using simulated data from 30 simulated patients (10 children, 10 adolescents, and 10 adults) generated with the Food and Drug Administration (FDA)–approved UVA/Padova simulator. A comparative analysis was conducted against state-of-the-art RL and ML models, evaluating performance using metrics such as TIR, hypoglycemia (<70 mg/dL), hyperglycemia (>180 mg/dL), and glycemic risk scores.

**Results:**

The PRIMO-FRL model achieved a robust overall TIR of 76.54%, with adults demonstrating the highest TIR at 81.48%, followed by children at 77.78% and adolescents at 70.37%. Importantly, the approach eliminated hypoglycemia, with 0.0% spent below 70 mg/dL across all cohorts, significantly outperforming existing methods. Mild hyperglycemia (180-250 mg/dL) was observed in adolescents (29.63%), children (22.22%), and adults (18.52%), with adults exhibiting the best control. Furthermore, the PRIMO-FRL approach consistently reduced glycemic risk scores, demonstrating improved safety and long-term stability in glucose regulation..

**Conclusions:**

Our findings highlight the potential of PRIMO-FRL as a transformative, privacy-preserving approach to personalized glycemic management. By integrating federated RL, this framework eliminates hypoglycemia, improves TIR, and preserves data privacy by decentralizing model training. Unlike traditional centralized approaches that require sharing sensitive health data, PRIMO-FRL leverages federated learning to keep patient data local, significantly reducing privacy risks while enabling adaptive and personalized glucose control. This multiobjective optimization strategy offers a scalable, secure, and clinically viable solution for real-world diabetes care. The ability to train personalized models across diverse populations without exposing raw data makes PRIMO-FRL well-suited for deployment in privacy-sensitive health care environments. These results pave the way for future clinical adoption, demonstrating the potential of privacy-preserving artificial intelligence in optimizing glycemic regulation while maintaining security, adaptability, and personalization.

## Introduction

### Background

Maintaining optimal blood glucose levels is a critical aspect of diabetes management, particularly for individuals with type 1 and advanced type 2 diabetes. Poor glycemic control can lead to severe short- and long-term complications, including hypoglycemia, hyperglycemia, cardiovascular disease, kidney failure, and neuropathy [1,2]. Hypoglycemia (<70 mg/dL) poses an immediate danger, causing confusion, seizures, and in extreme cases, coma or death. On the other hand, hyperglycemia (>180 mg/dL) increases the risk of long-term vascular and neurological complications. Given the life-threatening consequences of glucose fluctuations, maintaining time in range (TIR)—the percentage of time blood glucose remains between 70 and 180 mg/dL—has become a key metric in assessing diabetes management effectiveness.

In recent years, technological advancements have significantly improved glycemic management. Closed-loop insulin delivery systems, commonly known as artificial pancreas systems, use continuous glucose monitoring (CGM) sensors and insulin pumps to automate glucose regulation. These systems rely on control algorithms such as proportional-integral-derivative (PID) controllers and model predictive control (MPC) to adjust insulin dosing dynamically [3]. While these methods have enhanced automation, they remain limited in their ability to personalize treatment for individual patients due to variations in metabolism, diet, physical activity, and insulin sensitivity.

To address these limitations, machine learning (ML) and reinforcement learning (RL) approaches have emerged as promising alternatives, enabling data-driven and adaptive insulin dosing strategies [4-8]. However, these approaches still face significant challenges related to multiobjective optimization, privacy concerns, and generalizability across diverse patient populations.

### Research Gaps in Existing Methods

Traditional closed-loop control systems, such as PID- and MPC-based insulin delivery, rely on fixed mathematical models to regulate blood glucose levels [3]. These methods provide some level of automation but face limited adaptability to individual patient variations. Since they use predefined rules, they often fail to accommodate real-time metabolic fluctuations, making them less effective for personalized treatment. Moreover, these systems prioritize glucose stabilization but do not explicitly optimize other clinical factors, such as insulin efficiency and glycemic variability reduction [3].

ML techniques have improved glucose prediction and insulin recommendation by analyzing historical patient data. Supervised learning models, such as deep neural networks, have been used to forecast glucose trends based on CGM data [7,8]. While these models demonstrate strong predictive capabilities, they rely heavily on large datasets that may not always be available due to privacy regulations [9,10]. Furthermore, ML-based approaches lack decision-making capabilities, as they are designed for prediction rather than active control, making them insufficient for fully automated glucose regulation [8].

RL offers a more dynamic and adaptive solution, as it allows artificial intelligence (AI) models to learn optimal insulin dosing strategies through trial and error. Unlike traditional ML, RL does not require explicit supervision and can adjust insulin delivery based on real-time feedback from CGM data. Several RL-based frameworks have demonstrated improvements in personalized insulin dosing [4-7,11]. Recent work has also applied RL to personalize digital health interventions and optimize treatment recommendations in diabetes care [12,13]. However, existing RL methods face three major challenges:

1. Single-objective optimization: most RL models optimize only TIR but fail to balance other critical objectives such as reducing glycemic variability, preventing hypoglycemia, and minimizing insulin overuse [11,14].
2. Privacy and data security risks: standard RL models require centralized patient data for training, raising concerns about data privacy, security, and regulatory compliance [9,15,16].
3. Limited scalability and generalizability: traditional RL models often struggle to generalize across diverse patient populations, as they are typically trained on homogeneous datasets that may not reflect real-world variability in metabolic responses [17-19].

The limitations of existing glycemic management methods highlight the need for a privacy-preserving, scalable, and multiobjective learning framework. A truly effective system must:

1. Optimize multiple clinical goals: traditional models focus primarily on TIR, but optimal glycemic control requires simultaneously addressing insulin efficiency, hypoglycemia prevention, and glucose stability. A multiobjective RL approach is needed to balance these competing factors effectively.
2. Ensure data privacy without sacrificing learning efficiency: most AI-based systems rely on centralized training, which exposes sensitive patient data to security risks. Federated learning (FL) enables models to be trained locally on patient devices, ensuring privacy preservation while still allowing for robust learning. Recent studies have demonstrated the viability of FL to support privacy-preserving collaboration in health care, including frameworks tailored for clinical research [20,21].
3. Improve generalizability and scalability: existing methods often fail to adapt to diverse patient populations due to limited training data. A FL-based approach can leverage distributed patient data without centralizing information, thereby enhancing model robustness and adaptability.

### Proposed Solution: PRIMO-FRL

To address these challenges, we propose PRIMO-FRL (Privacy-Preserving Reinforcement Learning for Individualized Multi-Objective Glycemic Management Using Federated Reinforcement Learning). PRIMO-FRL integrates RL with FL to develop a secure, scalable, and adaptive system for personalized glycemic control.

PRIMO-FRL differs from existing approaches in several key ways. First, it incorporates multiobjective reward shaping, allowing the model to optimize not just TIR but also insulin efficiency, glycemic stability, and hypoglycemia prevention. Second, by leveraging FL, PRIMO-FRL enables decentralized training, ensuring that patient data remains local while still benefiting from collaborative learning across multiple devices. Finally, this approach improves scalability and generalizability by enabling models to learn from diverse patient populations without requiring direct data exchange.

### Key Contributions

This study introduces a novel PRIMO-FRL framework for glycemic management. The key contributions of this work are (1) development of a privacy-preserving RL framework that enables secure, decentralized model training without centralizing patient data, (2) integration of multiobjective optimization to balance TIR, insulin efficiency, glycemic stability, and hypoglycemia prevention, (3) scalable and adaptive learning mechanism that ensures generalizability across diverse patient populations using federated RL, and (4) secure and efficient model aggregation that prevents data breaches while allowing models to benefit from shared knowledge across distributed environments.

The aim of this study is to develop and validate PRIMO-FRL, a privacy-preserving federated RL framework for glycemic management that optimizes multiple clinical goals—maximizing TIR, reducing hypoglycemia and hyperglycemia, and minimizing glycemic risk—while ensuring patient data privacy through decentralized training.

## Methods

### Overview

Our proposed system, PRIMO-FRL, integrates FL and RL to develop a decentralized, privacy-preserving insulin optimization framework. Unlike traditional insulin delivery systems that rely on predefined algorithms or centralized AI models, PRIMO-FRL allows each patient device to locally train an RL agent, ensuring personalized adaptation while protecting sensitive medical data.

By leveraging federated RL, PRIMO-FRL enables a collaborative learning process where individual patient devices contribute to the improvement of a global insulin optimization model without sharing raw glucose and insulin data. This approach ensures that (1) personalized insulin dosing policies are developed based on patient-specific metabolic patterns, (2) multiobjective optimization is applied to balance glucose control, insulin efficiency, and safety, and (3) FL ensures privacy by keeping patient data local while still allowing collective model improvements.

This framework enables a privacy-compliant, scalable, and adaptive insulin therapy solution, addressing key challenges in modern glycemic management.

### Federated RL Workflow

**Figure 1.**
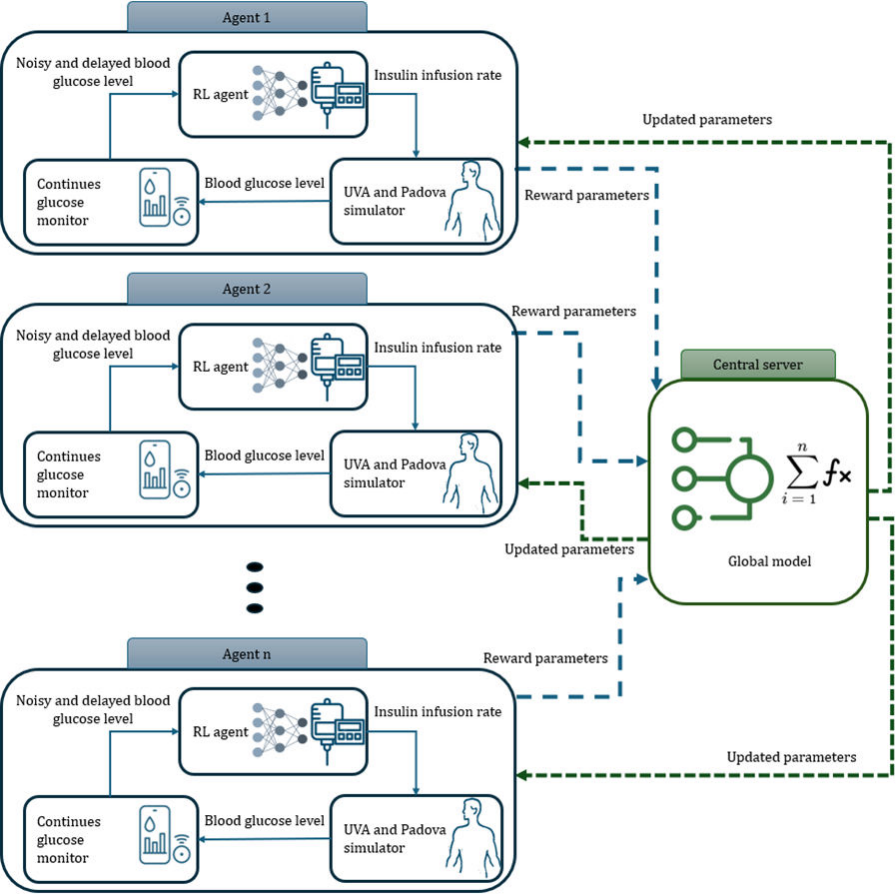
System architecture. RL: reinforcement learning.

Glycemic regulation in closed-loop insulin delivery systems can be framed as a decision-making process under uncertainty, modeled as a partially observable Markov decision process [8]. As shown in Figure 1, in this framework, an RL agent learns an insulin dosing policy through continuous interaction with the environment, receiving partial observations of the patient’s metabolic state.

The key components of the partially observable Markov decision process formulation in this context are defined as follows: (1)

1. State (s): the patient’s glucose level, insulin history, carbohydrate intake, and physiological parameters.
2. Action (a): the insulin dosage recommended by the RL agent.
3. Observation (o): real-time glucose readings from a continuous glucose monitoring (CGM) device, which are noisy and affected by sensor delays.
4. Reward (r): a multiobjective function balancing glucose control, insulin efficiency, and safety.

Since glucose metabolism is dynamic and influenced by past events, partially observable Markov decision process modeling allows the agent to make optimized insulin dosing decisions despite uncertainty in observations.

### System Components and FL Integration

PRIMO-FRL is structured around an FL framework in which multiple client devices (each representing an individual patient) collaboratively train a global model without centralizing patient data. The four main components of the system are as follows:

1. RL agents on client devices: each patient device runs a soft actor-critic (SAC) RL agent, which continuously learns an insulin dosing policy based on real-time glucose readings. SAC is particularly suitable for this application due to its ability to balance exploration and exploitation, ensuring that the agent learns optimal dosing strategies while adapting to patient-specific metabolic patterns [22].
2. UVA/Padova diabetes simulator [23,24]: a simulation tool for glucose-insulin dynamics in patients with type 1 diabetes, allowing agents to train in a controlled simulated environment.
3. Continuous glucose monitoring (CGM)/glucose sensor: each client device is connected to a CGM sensor, which provides glucose data with noise and delays to simulate real-world monitoring conditions.
4. FL coordination server: a central server acts as a federated coordinator, aggregating model updates from multiple patient devices while ensuring that raw glucose and insulin data remain private. The FL loop follows these steps: (1) each client device trains its RL agent locally; (2) instead of sharing patient data, the client sends model updates (reward parameters, policy weights, and gradients) to the central server; (3) the server aggregates the updates using federated averaging, adjusting for factors such as risk levels and patient variability; and (4) the updated global model is sent back to all clients, allowing each RL agent to benefit from knowledge gained across the network while maintaining patient privacy.

### Reward Optimization and Multiobjective Learning

A critical innovation in PRIMO-FRL is its multiobjective reward function, designed to balance competing goals in insulin therapy. It integrates components to encourage exploration (via policy entropy), penalize glucose deviations and insulin overuse, and reward glucose stability. The reward function balances these objectives through tunable coefficients.

A full breakdown of the mathematical formulation and hyperparameter tuning strategy is provided in Multimedia Appendix 1.

Our multiobjective reward function ensures that RL agents focus on multiple goals, such as maintaining safe blood glucose levels, preventing hypoglycemia, and optimizing insulin use. This personalized reward system, combined with the FL process managed by the central server, enables the system to provide better individualized care while benefiting from shared knowledge across all clients. In multiobjective optimization, conflicting goals—such as maintaining high TIR while minimizing insulin use—are handled through a carefully designed reward function that combines multiple clinical objectives using weighted coefficients (α, β, γ, and η). These weights were empirically tuned to reflect clinical priorities; for example, greater penalties are assigned to hypoglycemia and large glucose excursions than to moderate insulin use. This ensures that safety-critical outcomes are prioritized during training. In addition, the FL server plays a key role in balancing these trade-offs across the population. During the aggregation process, the server incorporates model updates from clients with diverse glycemic patterns and risk profiles. This distributed learning dynamic allows PRIMO-FRL to learn a globally balanced policy that generalizes well while still preserving local personalization. Future work may explore adaptive weighting strategies or Pareto front–based optimization to further refine trade-off resolution.

By integrating these advanced RL techniques with FL, our system not only enhances the personalization of glucose control but also ensures the privacy and security of patient data. This innovative approach addresses the critical challenges in diabetes management, paving the way for more effective and secure health care solutions.

### Ethical Considerations

This study did not involve human participants or identifiable personal data. All data were generated by the Food and Drug Administration (FDA)–approved UVA/Padova type 1 diabetes simulator.

## Results

### Overview

We conducted comprehensive experiments to evaluate the effectiveness of PRIMO-FRL with an entropy-based reward function for personalized blood glucose control. This section presents experimental results, highlighting the model’s performance across multiple patient cohorts and its effectiveness in achieving safe, adaptive, and privacy-preserving insulin optimization.

### Experimental Setup

#### Simulated Patient Data

Due to ethical, privacy, and logistical constraints in collecting real-world patient data, we used a validated simulation model for evaluation. The FDA-approved UVA/Padova simulator, a widely recognized tool for modeling glucose-insulin dynamics in patients with type 1 diabetes, was used to generate realistic, diverse patient data [19,25].

We simulated 30 simulated patients categorized into three groups: children, adolescents, and adults, with 10 patients per group. To promote realistic variability and support generalizability, these patients were randomly assigned to federated agents, ensuring heterogeneity in physiological characteristics such as insulin sensitivity, glucose absorption, and glycemic response. Each patient exhibited unique physiological characteristics to reflect diverse metabolic profiles. Data were generated over 5-day periods, including CGM readings recorded every 5 minutes and insulin dosages delivered at equivalent intervals. These simulated datasets provided a controlled and privacy-preserving environment to evaluate PRIMO-FRL’s performance.

#### Model Training and FL Implementation

The PRIMO-FRL framework was trained using federated reinforcement learning (FRL) principles. Each patient device independently trained a SAC RL agent, which optimized insulin dosing based on real-time glucose fluctuations.

The model parameters are as follows:

- Neural network architecture: 2 gated recurrent unit layers (128 hidden units each), followed by a fully connected output layer for insulin dosing decisions.
- Training details: 300 epochs, with a 5-day epoch length and a batch size of 256.
- FL aggregation: agents periodically transmitted model updates (policy gradients and reward parameters) to a central aggregation server, which performed federated averaging to update the global model.
- Reward function optimization: the entropy-based reward function incorporated glucose stability, insulin efficiency, and hypoglycemia prevention.

The trained models were validated using hold-out test data to ensure robust performance. To reduce the risk of overfitting, training incorporated several safeguards: (1) dropout layers were applied to neural network models, (2) model performance was periodically validated on hold-out simulated patients not used during training, and (3) early stopping was used based on plateaued improvements in validation metrics. Importantly, the FL framework trained agents across 30 diverse simulated patients spanning children, adolescents, and adults, promoting generalization by preventing overspecialization to any single cohort.

### Performance Evaluation

#### Glycemic Control Outcomes

PRIMO-FRL was evaluated based on key glycemic control metrics, particularly TIR, hypoglycemia prevention, and hyperglycemia reduction across all patient cohorts. The following summarizes the model’s performance across these clinical indicators:

1. TIR (70‐180 mg/dL): the TIR metric, a primary measure of glycemic management, showed that PRIMO-FRL effectively maintained blood glucose within the target range across all age groups: (1) adults achieved the highest TIR (81.48%), followed by children (77.78%), and adolescents (70.37%) and (2) overall TIR across all groups was 76.54% , surpassing clinical benchmarks for optimal glucose control.
2. Hypoglycemia (<70 mg/dL) prevention: a major highlight of PRIMO-FRL is its ability to completely eliminate hypoglycemia—0.0% time spent in hypoglycemia across all cohorts (0 out of 30 patients), demonstrating the framework's safety and effectiveness in preventing dangerously low blood glucose levels.
3. Hyperglycemia (>180 mg/dL) management: Mild hyperglycemia (180-250 mg/dL) was observed, particularly in younger patients. The adolescent group showed the highest incidence (29.63%), followed by children (22.22%), and adults (18.52%). These findings are consistent with known age-related glucose variability.
4. Severe hyperglycemia (>250 mg/dL): no severe hyperglycemia was observed in any cohort, demonstrating the system’s robustness in managing extreme glucose excursions.

Table 1 provides a detailed breakdown of glycemic control performance.

**Table 1.** Aggregated glycemic control results across patient groups. Percentages represent the average proportion of simulation time spent in each glucose range per group (N=10 simulated patients per group).

Patient group	<50 mg/dL, %	50‐70 mg/dL, %	70‐180 mg/dL, %	180‐250 mg/dL, %	>250 mg/dL, %
Child	0.0	0.0	77.78	22.22	0.0
Adolescent	0.0	0.0	70.37	29.63	0.0
Adult	0.0	0.0	81.48	18.52	0.0
Overall	0.0	0.0	76.54	23.46	0.0

#### Comparative Analysis With Existing Studies

To contextualize PRIMO-FRL’s effectiveness, we compared its results with prior ML- and RL-based blood glucose regulation methods. A detailed comparison with prior ML and RL methods is available in Multimedia Appendix 2.

#### Key Observations and Improvements

The following key findings highlight how PRIMO-FRL improves upon existing methods across safety, glycemic control, and clinical effectiveness:

1. Superior safety*:* PRIMO-FRL eliminated hypoglycemia entirely, outperforming prior approaches such as RL-Scratch in [8], which reported a 0.73% hypoglycemia incidence.
2. Strong TIR performance: the overall TIR of 76.54% aligns with or surpasses other RL-based methods (eg,~73% in [5]).
3. Effective hyperglycemia control: mild hyperglycemia (180‐250 mg/dL) was effectively managed, and severe hyperglycemia (>250 mg/dL) was avoided in all groups.

### Risk Improvement and Stability Analysis

**Figure 2.**
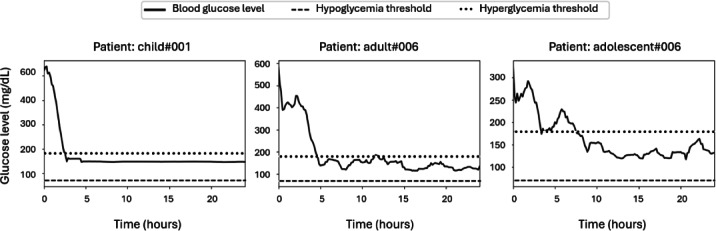
Twenty-four–hour glucose level trajectories for representative patients.

Figure 2 presents the 24-hour blood glucose trajectories for 3 representative patients. The blue line represents blood glucose levels, while the dashed orange and red lines indicate the hypoglycemia (70 mg/dL) and hyperglycemia (180 mg/dL) thresholds, respectively. The key insights from this figure include the following:

1. Effective glucose control: the PRIMO-FRL approach successfully maintains glucose levels within safe physiological ranges, preventing both severe hypoglycemia and hyperglycemia.
2. Stable glycemic response: patient child#001 starts with high glucose levels, which gradually decline and stabilize. Similarly, adult#006 and adolescent#006 exhibit relatively stable glucose trajectories, with fewer extreme fluctuations.
3. Reduced hyperglycemia episodes: the glucose trends suggest that the FRL model helps mitigate prolonged hyperglycemia, particularly for adolescent#006, who demonstrates a downward trend toward normal glucose levels.

**Figure 3.**
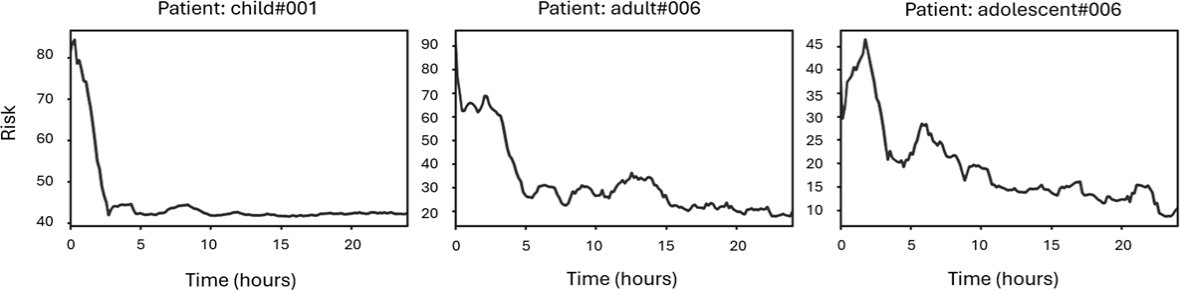
Twenty-four–hour risk improvement for representative patients.

Figure 3 presents the risk trajectories for the same patients over 24 hours. The risk score (y-axis) quantifies the likelihood of adverse outcomes due to hypoglycemia or hyperglycemia, while the x-axis represents time. The key observations include:

1. Consistent risk reduction: across all 3 patients, the PRIMO-FRL approach significantly lowers risk scores over time, demonstrating its ability to penalize extreme glucose deviations while reinforcing stable glucose management.
2. Reduced glycemic variability: patients experience a noticeable decline in risk levels. For example, child#001 initially exhibits high risk, which then stabilizes at a lower level, indicating a reduced likelihood of hypoglycemic or hyperglycemic events. Similarly, adult#006 and adolescent#006 show smoother, sustained risk reductions, suggesting better glucose regulation.
3. Improved stability: over time, risk scores not only decrease but also stabilize, as evidenced by the flattening trends in later hours. This stability highlights the FRL approach’s effectiveness in providing long-term consistency in glucose management.

These findings confirm that the PRIMO-FRL approach effectively reduces patient risk while ensuring a more stable and safe glucose control mechanism.

## Discussion

### Principal Findings

In this work, we proposed a novel FRL framework, PRIMO-FRL, for personalized blood glucose control, incorporating an entropy-based reward function to enhance adaptability and privacy preservation. Our approach addresses key challenges in diabetes management, including variability in patient responses, data privacy concerns, and the need for robust, individualized treatment strategies. By leveraging FL, PRIMO-FRL ensures that sensitive patient data remains local, thus safeguarding privacy while benefiting from the diversity of data across patients.

Experimental evaluations using validated simulation models demonstrated the effectiveness of our approach, achieving significant improvements in risk scores, time in the euglycemic range, and glucose stability across diverse patient categories. The entropy-based reward function encouraged policy exploration, effectively balancing glucose regulation, insulin usage, and glucose variability. Notably, these improvements were achieved without increasing insulin use, underlining the system’s efficiency and safety.

### Limitations

While PRIMO-FRL offers several advantages, certain limitations must be acknowledged, and future directions should aim to address these challenges.

#### Simulation Model Dependence

The experimental results were obtained using validated simulation models. While these models provide valuable insights, real-world validation is essential to confirm the efficacy and safety of the framework in clinical settings. Although the model was trained on simulated data, we took precautions to avoid overfitting through dropout, early stopping, and evaluation on held-out patient profiles. Moreover, the federated structure naturally supports generalization, as it forces the model to adapt across distributed, heterogeneous patient agents. Future work involving real-world clinical validation will further evaluate model robustness and generalizability to unseen metabolic patterns. Integration with real-world CGM systems would likely occur through edge devices (eg, smartphones or insulin pump controllers), which would enable real-time data processing and model updates using FL without compromising data privacy.

#### Communication Overheads

FL relies on transferring model updates between a central server and local devices. Although we aimed to minimize data exchange, communication overheads could still be a bottleneck in low-bandwidth or high-latency environments. Another potential challenge in real-world deployment is the communication overhead introduced by FL. PRIMO-FRL relies on periodic transmission of model updates (eg, policy gradients and reward parameters) between client devices and the central aggregator. In environments with limited bandwidth or high latency—such as rural areas or underresourced clinics—this could hinder real-time synchronization and degrade performance. To mitigate this, future iterations of PRIMO-FRL may incorporate model compression techniques, sparse update schemes, or asynchronous FL approaches to reduce communication costs while maintaining training efficiency and model accuracy.

#### Patient Heterogeneity

Individual patient variability is high in diabetes management. While FL accommodates diverse patient data, additional personalization strategies or more extensive data might be necessary for complex cases with outlier metabolic profiles.

#### Privacy and Security Considerations

While FL enhances privacy by keeping raw patient data local, it is not immune to adversarial threats. Techniques such as gradient inversion attacks could potentially reconstruct sensitive information from shared model updates, and adversarial participants could perform model poisoning to degrade global performance. To mitigate these risks, future iterations of PRIMO-FRL could incorporate privacy-preserving techniques such as differential privacy, secure multiparty computation, or robust aggregation algorithms (eg, Krum or Trimmed Mean). In addition, anomaly detection methods can help identify and exclude malicious client updates, further strengthening system resilience.

### Comparison With Prior Work

Prior research in AI-driven glycemic management has explored CGM systems, artificial pancreas technologies, and RL-based insulin dosing [2,3]. However, most existing approaches rely on centralized data collection, raising serious concerns about patient privacy, security, and regulatory compliance [9].

PRIMO-FRL offers a fundamental shift from these centralized approaches by leveraging federated RL, which ensures that raw patient data never leaves local devices. This privacy-preserving architecture aligns with recent advancements in decentralized learning paradigms but extends prior work by integrating a multiobjective entropy-based reward function specifically tailored for personalized insulin optimization. Unlike traditional RL methods that require pooled, centrally stored data [4,5,11], PRIMO-FRL achieves comparable or superior glycemic control performance while preserving patient confidentiality.

In addition, PRIMO-FRL addresses key limitations in existing RL-based insulin dosing systems, which often prioritize single-objective optimization (eg, maximizing TIR) [8,11]. By contrast, PRIMO-FRL explicitly optimizes multiple clinical goals, including hypoglycemia prevention, glucose stability, and insulin efficiency, making it better suited for real-world applications where trade-offs between multiple factors are necessary.

Compared to traditional diabetes management strategies, such as manual insulin dosing guided by fixed algorithms or carbohydrate counting, PRIMO-FRL offers a more adaptive and personalized approach. Standard insulin delivery methods, such as manual basal-bolus regimens or PID- or MPC-based closed-loop systems, are limited by fixed-rule control and lack of adaptability to individual metabolic responses. In contrast, PRIMO-FRL dynamically adjusts insulin dosing through real-time learning and explicitly incorporates clinical goals—including hypoglycemia avoidance, glycemic stability, and insulin efficiency—into its optimization framework. This alignment with current clinical best practices, combined with its ability to personalize care while preserving privacy, positions PRIMO-FRL as a clinically relevant advancement in AI-driven diabetes management.

### Conclusions

Our findings underscore PRIMO-FRL as a transformative, privacy-preserving framework for glycemic management. By eliminating hypoglycemia, improving TIR, and leveraging federated RL, PRIMO-FRL offers a scalable, secure, and multiobjective optimization strategy for real-world diabetes care.

Beyond diabetes management, the principles demonstrated in PRIMO-FRL have broader implications for other chronic disease management applications, where personalized, privacy-preserving, AI-driven decision-making is critical. Future work will focus on clinical trials, real-world deployment, and further optimization of the FL process to enhance efficiency, scalability, and adaptability across diverse patient populations.

Through its integration of RL, FL, and multiobjective optimization, PRIMO-FRL represents a next-generation approach to AI-driven personalized medicine, paving the way for safer, more adaptive, and privacy-compliant digital health solutions. While PRIMO-FRL demonstrates strong performance in glucose regulation, interpretability remains an important consideration for clinical acceptance. In practice, health care providers often require insight into the rationale behind AI-generated decisions, especially in high-risk domains like insulin dosing. Future work will explore methods to improve the explainability of RL agents, such as incorporating attention-based visualization of decision factors, generating counterfactual scenarios to highlight what influenced a particular dosing choice, or applying inherently interpretable policy architectures. Enhancing transparency will support shared decision-making and promote clinician confidence in AI-assisted care.

## Supplementary material

10.2196/72874 Multimedia Appendix 1 Detailed reward function.

10.2196/72874 Multimedia Appendix 2 Comparative performance analysis with baseline machine learning and reinforcement learning models.

## Abbreviations

AI: artificial intelligence
CGM: continuous glucose monitoring
FDA: Food and Drug Administration
FL: federated learning
FRL: federated reinforcement learning
ML: machine learning
MPC: model predictive control
PID: proportional-integral-derivative
PRIMO-FRL: Privacy-Preserving Reinforcement Learning for Individualized Multi-Objective Glycemic Management Using Federated Reinforcement Learning
RL: reinforcement learning
SAC: soft actor-critic
TIR: time in range
