# Supplementary material for: Privacy-Preserving Glycemic Management in Type 1 Diabetes: Development and Validation of a Multiobjective Federated Reinforcement Learning Framework
Source: JMIR Diabetes. 2025 Jul 4;10:e72874. doi: 10.2196/72874 (PMC12248133; doi:10.2196/72874)
Supplement: Multimedia Appendix 1 [file diabetes-v10-e72874-s001.docx]

**Multimedia Appendix 1. Detailed Reward Function**

The multi-objective reward function used in PRIMO-FRL is defined as:

$$R=\alpha H(\pi)-\beta\frac{\left( G-125 \right)^{2}}{100}-\gamma\frac{U}{10}+\eta.\frac{1}{\sqrt{1+Var(G)}}$$

Where:

- $H(\pi)$: Entropy of the policy—encouraging exploration and preventing premature convergence.
- $\left( G-125 \right)^{2}$: Squared discrepancy from target glucose level—Penalizes deviations from the target optimal glucose level (125 mg/dL), promoting stable glucose control.
- $U$: insulin infusion rate term—Penalizes excessive insulin dosing to prevent hypoglycemia.
- $Var\left( G \right):$Variance of Glucose Levels—Rewards stability in glucose levels by considering the variance of recent glucose readings.
- The coefficients (alpha, beta, gamma, eta) are hyperparameters tuned to balance these objectives, ensuring that the RL agent learns an effective insulin delivery policy.
