# Supplementary material for: Privacy-Preserving Glycemic Management in Type 1 Diabetes: Development and Validation of a Multiobjective Federated Reinforcement Learning Framework
Source: JMIR Diabetes. 2025 Jul 4;10:e72874. doi: 10.2196/72874 (PMC12248133; doi:10.2196/72874)
Supplement: Multimedia Appendix 2 [file diabetes-v10-e72874-s002.docx]

### Multimedia Appendix 2. Comparative performance analysis with baseline ML and RL models

Table 2: Performance Comparison with Existing ML and RL Approaches

| Study | Method | TIR (70–180 mg/dL) | Time <70 mg/dL | Time >180 mg/dL |
| --- | --- | --- | --- | --- |
| [5] | RL | ~73% | Not specified | Not specified |
| [11] | Multi-Step Deep RL | ~85.62% | Not specified | Not specified |
| [8] | Deep RL (RL-Scratch) | 72.68% | 0.73% | 26.17% |
| PRIMO-FRL(This Work) | Federated RL (FRL) | 77.78% (Child)  70.37% (Adol.)  81.48% (Adult)  76.54% (Overall) | 0.0% for all groups | 22.22% (Child) 29.63% (Adol.) 18.52% (Adult) |
